# Does preoperative multidisciplinary team assessment of high-risk patients improve the safety and outcomes of patients undergoing surgery?

**DOI:** 10.1186/s12871-023-02394-5

**Published:** 2024-01-02

**Authors:** B. I. Kuiper, L.M.J. Janssen, K. S. Versteeg, B. L. ten Tusscher, J. I. van der Spoel, W. D. Lubbers, G. Kazemier, S. A. Loer, P. Schober, V. P. van Halm

**Affiliations:** 1 Department of Surgery, Amsterdam UMC location VUmc, Amsterdam, The Netherlands; 2 Cancer Center Amsterdam, Amsterdam, The Netherlands; 3 Department of Anesthesiology, Amsterdam UMC location VUmc, De Boelelaan 1117, Amsterdam, 1081 HV The Netherlands; 4 Department of Internal medicine, section geriatrics, Amsterdam UMC location VUmc, Amsterdam, The Netherlands; 5 Department of Intensive Care Medicine, Amsterdam UMC location VUmc, Amsterdam, The Netherlands; 6 Department of Cardiology, Amsterdam UMC location VUmc, Amsterdam, The Netherlands

**Keywords:** Multidisciplinary team, Preoperative consultation, Anesthesia, Frailty, High-risk surgery

## Abstract

**Background:**

International guidelines recommend preoperative multidisciplinary team (MDT) assessment for high-risk surgical patients. Preoperative MDT meetings can help to improve surgical care, but there is little evidence on whether they improve patient outcomes.

**Methods:**

This paper aims to share our experience of MDT meetings for high-risk surgical patients to underline their added value to the current standard of care. An observational study of a retrospective cohort of preoperative high-risk MDT meetings of a tertiary referral hospital between January 2015 and December 2020. For 249 patients the outcomes preoperative data, MDT decisions, and patient outcomes were collected from electronic health records.

**Main results:**

A total of 249 patients were discussed at high-risk MDT meetings. Most of the patients (97%) were assessed as having an American Society of Anesthesiology score ≥ 3, and 219 (88%) had a European Society of Cardiology and European Society of Anaesthesiology risk score of intermediate or high. After MDT assessment, 154 (62%) were directly approved for surgery, and 39 (16%) were considered ineligible for surgery. The remaining 56 (23%) patients underwent additional assessments before reconsideration at a high-risk MDT meeting. The main reason for patients being discussed at the high-risk MDT meeting was to assess the risk-benefit ratio of surgery. Ultimately, 184 (74%) patients underwent surgery. Of the operated patients, 122 (66%) did not have a major complication in the postoperative period, and 149 patients (81%) were alive after one year.

**Conclusions:**

This cohort study shows the vulnerability and complexity of high-risk patients but also shows that the use of an MDT assessment contributes too improved peri- and postoperative treatment strategies in high-risk patients. Most patients underwent surgery after careful risk assessment and, if deemed necessary, preoperative and perioperative treatment optimization to reduce their risk.

**Supplementary Information:**

The online version contains supplementary material available at 10.1186/s12871-023-02394-5.

## Background

Physicians are increasingly being challenged with more complex surgeries due to technical innovations and more complex patients due to expanding indications in a population with increasing age and frailty [1–4]. The risk of serious perioperative or postoperative complications can be disproportionately high for patients with extensive multimorbidity or frailty compared to the potential benefits they might obtain from surgery. Complications can lead to increased healthcare costs and long-term reductions in quality of life, functionality, and mortality [5, 6]. To prevent unnecessary increases in patient burden and to limit the strain on available resources, consideration of the risk-benefit ratio of surgery is needed [7].

Despite the availability of prediction tools [8], studies have shown that senior and resident anesthesiologists have difficulties to estimate surgical risks based on preoperative data [9]. Furthermore, despite the potential utility of prediction-based research, the use of risk assessment tools for individual patients remains challenging [10]. For several years, international guidelines such as those provided by the American College of Cardiology/American Heart Association (ACC/AHA), the European Society of Anesthesiology, and the European Society of Cardiology (ESA/ECS), have recommended a preoperative multidisciplinary team (MDT) assessment in addition to the use of prediction tools for high-risk patients [11, 12]. The purpose of an MDT assessment for high-risk patients with multiple relevant healthcare physicians present is to facilitate discussion and to decide on the best treatment for the individual patient [13]. The goal of an MDT meeting to reach a consensus on optimal care has been described in more detail for several diseases but is probably best established in cancer care [14, 15]. Although it may seem likely that a preoperative MDT assessment improves patient care and outcomes, evidence for its value in high-risk surgical patients is limited, and MDT meetings are still infrequently used for the evaluation of high-risk patients [7]. The present study aimed to share our experience of holding preoperative MDT meetings for high-risk surgical patients to endorse the use of such meetings.

## Methods

### Study design and setting

This retrospective observational study includes all patients who were discussed at an MDT meeting between January 2015 and December 2020. Our institution, Amsterdam University Medical Center, VUmc, an academic and tertiary referral hospital in the Netherlands, hosted weekly preoperative high-risk MDT meetings over this period. In this study we assimilate the results of our MDT meeting to an international standardized pre-operative risk calculator with the aim to demonstrate if the MDT is able to assess high-risk patients.

### Participants

Patients with advanced systemic disease, advanced age, or limited functional capacity, in whom there was severe doubt that potential benefits would outweigh the surgical risk, were included. At the start of implementation, cases were mainly selected by the anesthesiologist during preoperative consultation in the outpatient clinic. Over time, surgical specialists have become more active in the selection of cases. There was no hard objective score such as the use of a risk calculator to select with patient should be discussed during the MDT. The inclusion of the patient was mainly based on experience of the physician.

### Multidisciplinary team meeting

This MDT meeting for high-risk patients was a result of a health quality improvement project to create a better communication between different physicians in case of complex and high-risk patients. Most of the patients are technically able to receive anesthesia, however most of the time the risks after the anesthesia determine the balance between risks and benefits. To make sure that every engaged physician agreed on performing the surgery, and the team already made treatment strategies for the postoperative period; this MDT was designed. The meeting was organized weekly by the Department of Anesthesiology (chairman) on a set time and place. Patients were registered to the meeting by the anesthesiologist, who performed the preoperative screening, and later in this project also by the surgeon. The primary aim of the meeting was to consider the risk-benefit ratio of surgery for each case individually. The risk-benefit ratio of surgery was mainly a qualitative discussion based on the combined expertise of the different present physicians. Furthermore, other topics discussed during the meeting included the need for an extra preoperative consultation due to comorbidities, prehabilitation, anesthetic techniques, the possibility of alternative treatment, and the need for postoperative treatment in a medium or intensive care unit.

When the MDT meetings were introduced in 2015, the team consisted of an anesthesiologist, cardiologist, intensivist, and treating physician. In 2017, a geriatrician joined the regular MDT meetings. Other physicians, such as pulmonologists, nephrologists, or neurologists, were invited to attend the meetings when indicated. The chairman monitored the time per patient, a maximum of ten minutes per patient was agreed on, to make sure discussions were efficient and to the point.

During the MDT meetings, the treating physician would present the patient including medical history, relevant comorbidities, performance status, the scheduled surgery and possible concerns of the risks. Thereafter present physicians were asked to elucidate the comorbidity and related prognoses caused by that specific comorbidity when relevant., The chairman summarized all the information en formulates and organized that the discussion resulted in consensus-based advice: (1) approval for surgery, (2) reassessment or (3) ineligibility for surgery. When reassessment was suggested this could be because of the need for extra consultation of, by example, a pulmonologist for the pulmonary performance or a cardiologist for the cardiac function. Other reasons for a reassessment was when the team advised prehabilitation for improvement of the performance status of the patient. After this advice was performed, the patient was rediscussed during the next meeting for a final decision of approval or ineligibility. The conclusion and advice of the MDT were noted in the electronic medical records of the patient, together with the considerations of the meeting.

### Patient characteristics

For this study demographic and clinical data were retrospectively collected from the electronic medical records. The baseline characteristics included age, sex, weight, height, body mass index, presence of polypharmacy defined as ≥ five medicinal products, Charlson Comorbidity Index (CCI), American Society of Anesthesiology (ASA) score, and ESC/ESA risk score. The CCI was calculated for each patient by the investigator (BK) [16]. A score for frailty was not used in this study due to the fact that there was too much missing data for the patients before 2017, the year the geriatrician was added to the physicians of the MDT. Intervention was defined as either diagnosis or treatment. Furthermore, the intention to intervene was defined as either curative or palliative.

### Outcome

The primary outcome of the present study was the advice of the MDT meeting: i.e., (1) approval for surgery, (2) reassessment or (3) ineligibility for surgery. This advice was linked with the outcome of the risk of severe complications and death according to the American College of Surgeons (ACS) National Surgical Quality Improvement Program (NSQIP) surgical risk calculator [17] to investigate the ability of the MDT to assess vulnerable patients.the ACS NSQIP surgical risk calculator was used for each patient by the investigator (BK) and retrospective calculated for all included patients. The ACS NSQIP was used on indication during the MDT, unfortunately this was not routinely registered in the electronic medical record. The secondary outcomes were postoperative complications within 30 days, graded according to Clavien and Dindo [18] (Supplementary Table A). A Clavien-Dindo grade of 3a or higher is considered clinically relevant because the patient needs a new intervention under general anesthesia or is admitted to the intensive care unit (ICU). Other secondary outcomes were the length of stay in the ICU and hospital, the location of discharge, and one year mortality.

### Statistical analysis

All the results of this study were shown by the primary outcome (1) approval for surgery, (2) reassessment, or (3) ineligible for surgery. Continuous variables are displayed as the mean with standard deviation or median with interquartile range (IQR) based on the distribution. Categorical variables are presented as counts and percentages. To demonstrate the vulnerability of the different groups, baseline characteristics were compared using the Pearson chi-square test and a one-way analysis of variance, where appropriate. The Kaplan-Meier method was used to depict survival in different groups. Statistical analyses were performed using SPSS® version 26.0 (IBM®, Armonk, NY, USA).

### Ethical considerations

The local Medical Ethics Review Committee of the Amsterdam UMC location VUmc (address: De Boelelaan 1109, room 08 A-08, PO-box 7057, Postal code 1081 HV in Amsterdam, the Netherlands), presided by prof. dr. J.A.M. van der Post, decided on July 7th 2021, after careful consideration, to grant the present study a waiver because it was seen as part of internal quality inquiry as described in the legislation concerning “healthcare quality, complaints and disputes act” (known in the Netherlands as: “Wet Kwaliteit, klachten en geschillen zorg).

## Results

From January 2015 to December 2020, 249 patients were assessed at the high-risk MDT meeting of the Amsterdam University Medical Center, VUmc. A total of 253 patients were enrolled, but four cases were never discussed in the high-risk MDT meeting; two patients died before being discussed, one patient decided not to proceed with surgery before the MDT, and in one patient, the surgeon decided to refrain from surgery before the MDT because the risk of complications or even death was considered too high. The number of patients discussed, compared to the number of performed elective surgeries in our hospital, were: 37/10,829, 31/9786, 37/13,896, 38/12,560, 51/15,101, 55/13,009 in 2015, 2016, 2017, 2018, 2019, and 2020, respectively. The increasing number of MDT’s is due to the improved consciousness of the MDT.

The demographic characteristics of the patients are presented in Table 1. The median age of our population was 71 years (IQR 61–78), and 242 patients (97.2%) had an ASA score ≥ 3. Most interventions (97.2%) were proposed in the context of treatment, and only a small portion were diagnostic. Furthermore, 219 (88%) procedures were classified as intermediate- or high-risk according to the ESC/ESA risk score.

**Table 1:** Demographic characteristics patients

			All patients n = 249	Approval for surgery n=154 (62%)	Re-assessment n=56 (23%)	Ineligible for surgery n=39 (16%)	p-value
Sex (male) (%)			152 (61)	95 (62)	31 (55)	26 (67)	*0.533*
Age (SD)			68.7 (14)	66.7 (15)	72.2 (13)	72.3 (9)	*0.883*
American Society of Anesthesiology (ASA) score (%)		2	7 (3)	7 (5)	0	0	0.001*
		3	135 (54)	93 (60)	31 (55)	11 (28)	
		4	107 (43)	54 (35)	25 (45)	28 (72)	
Charlson Comorbidity index (CCI) (SD)			6.5 (3)	6.1 (3)	7 (3)	7.5 (3)	*0.736*
Body Mass Index (BMI) (SD)			26.6 (7)	26.8 (6)	26.1 (7)	26.1 (7)	*0.374*
Number of patients with polyfarmacy~ (%)			201 (81)	127 (83)	40 (71)	34 (87)	*0.505*
8 missing data				3 missing data	5 missing data		
Context of intervention within treatment (%)			242 (97)	151 (98)	54 (96)	37 (95)	*0.521*
Oncological indication (%)			104 (42)	69 (45)	22 (39)	13 (33)	*0.393*
Palliative nature (%)			18 (7)	12 (8)	4 (7)	2 (5)	*0.848*
ESC/ESA risk score procedure (%)	Low		30 (12)	15 (10)	7 (113)	8 (21)	0.402**
	Intermediate		157 (63)	101 (66)	33 (59)	23 (59)	
	High		62 (25)	38 (24)	16 (29)	8 (25)	

~ ≥5 medicinal products

* p-value illustrates ASA 2 compared to ASA 3 and ASA 4 between the different stratified groups

**p-value illustrates the low risk score compared to the intermediate and high risk score between the different stratified groups

**Fig. 1:**
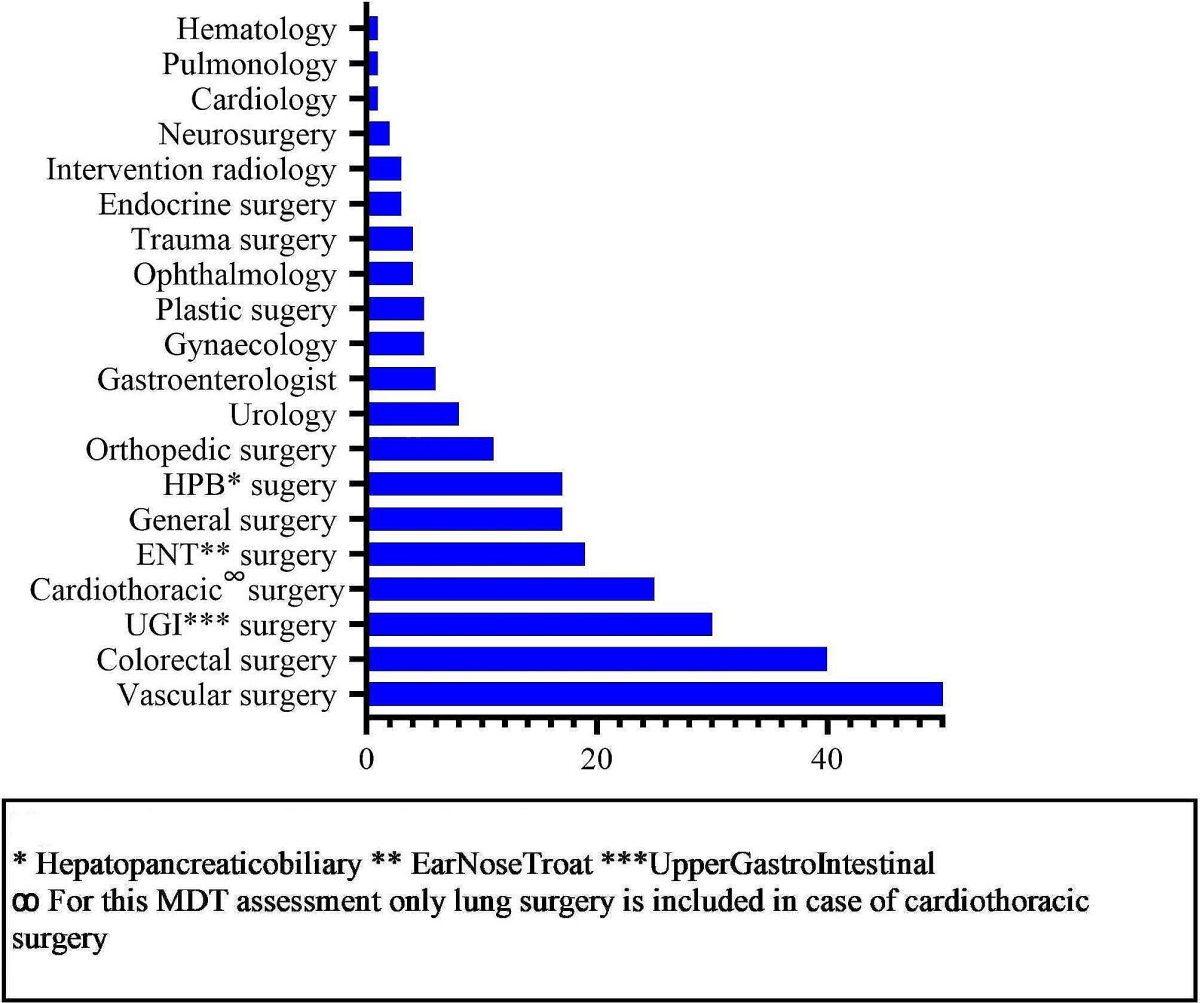
Expertise of the surgical physician

The most common reason for presenting patients in the MDT meeting was the presence of severe comorbidity or frailty impacting the tolerability of the intervention and/or anesthesia and/or the anticipated risk of a complicated postoperative trajectory. Another frequent reason for presenting a case was to discuss a rarely performed and/or highly complex intervention. In most patients, there was more than one reason for discussing their case in the MDT. The departments of the surgical physicians that introduced patients at the MDT meeting are summarized in Fig. 1. A median of four (IQR 3–5) different physicians were present during the MDT meeting, ranging up to nine. Detail on the involvement of the cardiologist, pulmonologist, and geriatrician in the preoperative period is listed in Supplementary Table B. In 44 (17.6%) patients, these physicians were involved based on the advice for additional assessment instigated during the MDT meeting. Other reasons for the involvement of these physicians were based on the 2014 ESC/ESA Guidelines [12], as indicated preoperatively by an anesthesiologist.

### Results high-risk multidisciplinary team assessments

**Fig. 2:**
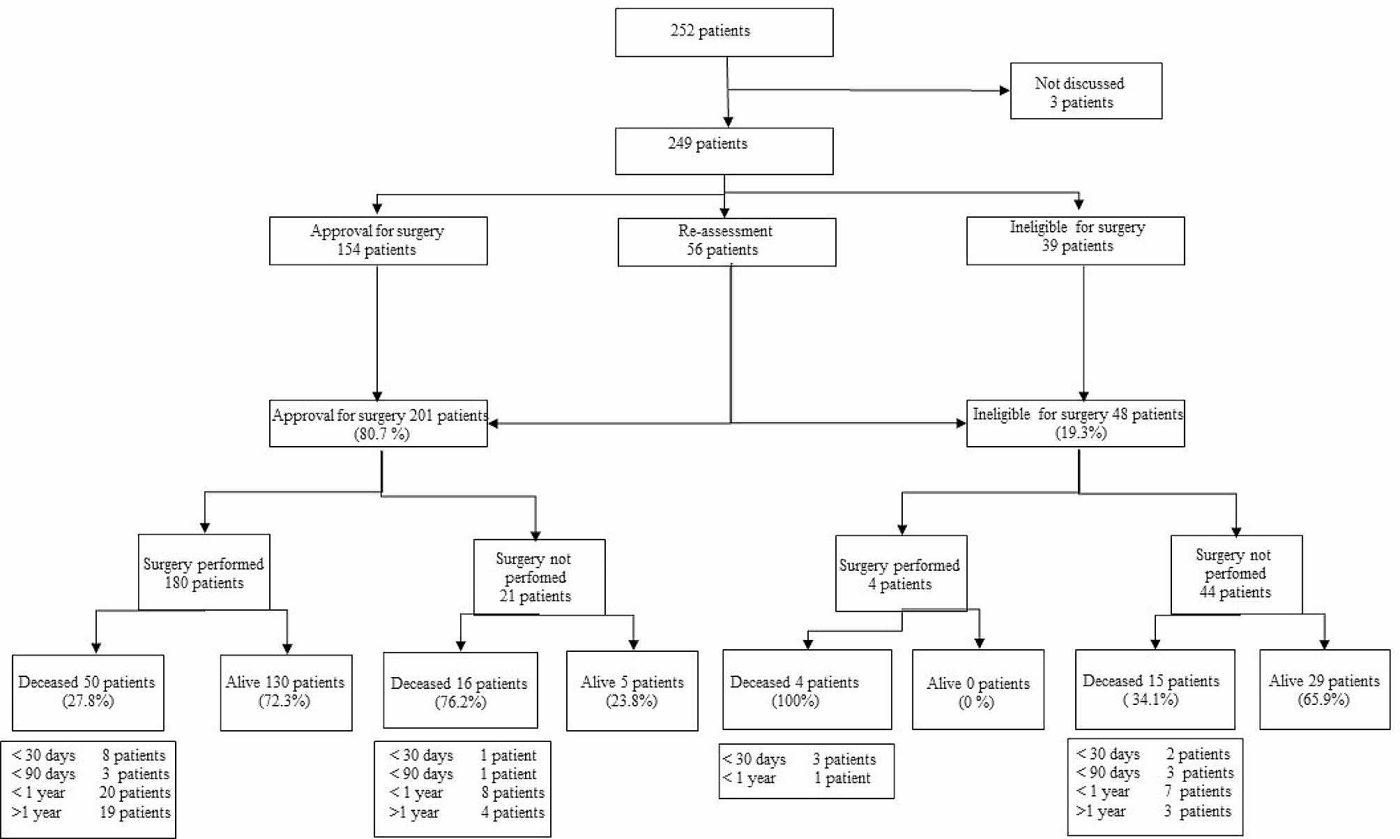
Outcome MDT meeting including performed surgery and overall survival

The results of the MDT meetings are summarized in Fig. 2. After primary assessment, direct approval for surgery was given to 154 (61.8%) patients, reassessment was suggested in 56 (22.5%) patients, and 39 (15.7%) patients were considered ineligible for surgery. Of the 56 reassessed patients, 47 (18.9%) were approved for surgery at the second instance, and eight (3.2%) patients were definitively thought to be ineligible for surgery. Of all the patients discussed, 201 (80.7%) were finally approved for surgery.

The most common reason for patients being considered ineligible for surgery was the inability to return to premorbid function because of the inability to recover during the postoperative period because of severe cardiovascular or pulmonary comorbidities or a lack of resilience. Another common reason was if they could be treated with a less invasive alternative with a more acceptable risk-benefit ratio (Supplementary Table C).

As shown in Table 2, the outcomes of the high-risk MDT were combined with the outcomes of the ACS NSQIP surgical risk calculator. The risk of severe complications and death calculated by the ACS NSQIP surgical risk calculator is rendered by the three different MDT recommendations (approved, reassessed, or ineligible). The input parameters for the ACS NSQIP surgical risk calculator are summarized in Supplementary Table D and rendered using the three different MDT recommendations. When the ACS NSQIP surgical risk calculator was used to calculate the risk of severe complications and death within 30-days using the data of the study population 84% and 78% of the patients, respectively, had an above-average chance of these outcomes.

**Table 2:** Output ACS NSQIP surgical risk calculator subdivided by the three different advices of the MDT assessment

		ACS NSQIP surgical risk calculator calculated risk on severe complication*			ACS NSQIP surgical risk calculator calculated risk of death		
MDT outcome		Below average	Equal average	Above average	Below average	Equal average	Above average
	Approval for surgery (%)	6 (4)	26 (17)	115 (75)	11 (7)	24 (16)	106 (69)
	Reassessment (%)	1 (2)	2 (3)	51 (91)	0	5 (9)	46 (82)
	Ineligible for surgery (%)	0	3 (7)	33 (85)	0	2 (5)	33 (84)

*(Cardiac arrest, myocardial infarction, pneumonia, progressive renal insufficiency, acute renal failure, PE, DVT, return to operating room, deep incisional SSI, organ space SSI, systemic sepsis, unplanned intubation, UTI, wound disruption)

### Interventions and postoperative outcomes

Of the 201 patients approved for surgery, 180 (89.6%) underwent a diagnostic or therapeutic intervention under anesthesia after the MDT meeting. Another four patients who were considered ineligible for surgery by the MDT underwent an intervention. Three patients underwent emergency surgery in the palliative phase to improve their quality of life. In the fourth case, the anesthetic technique was changed from sedation to general anesthesia due to the necessity of intervention. Three of these four patients experienced severe postoperative complications.

Of the 184 patients who underwent surgery, 59 (32.7%) had a complication grade of ≥ 3a recorded. Seventeen (9.4%) patients developed postoperative delirium. The ICU length of stay was two days (median, range 1–75), and the medium care unit length of stay was one day (median, range 1–17). Twelve patients were readmitted to the ICU during their hospital stay. In the operated patients, the 30-day mortality was 4.4%. The median length of hospital stay was 6.5 days (3.75-12). A total of 131 (71.2%) patients were discharged directly to their homes, 35 (19%) patients were discharged to a nursing or revalidation home, five (2.7%) patients died in hospital, and 13 (7.1%) patients had no data recorded regarding their discharge destination.

### One year mortality

The one year mortality in patients who underwent surgery was 19% (35/184 patients). In the group of patients considered ineligible for surgery, 33.8% (22/65) of the patients died during the one year follow-up period. All four patients who underwent surgery despite being considered ineligible for surgery died within the first year.

**Fig. 3:**
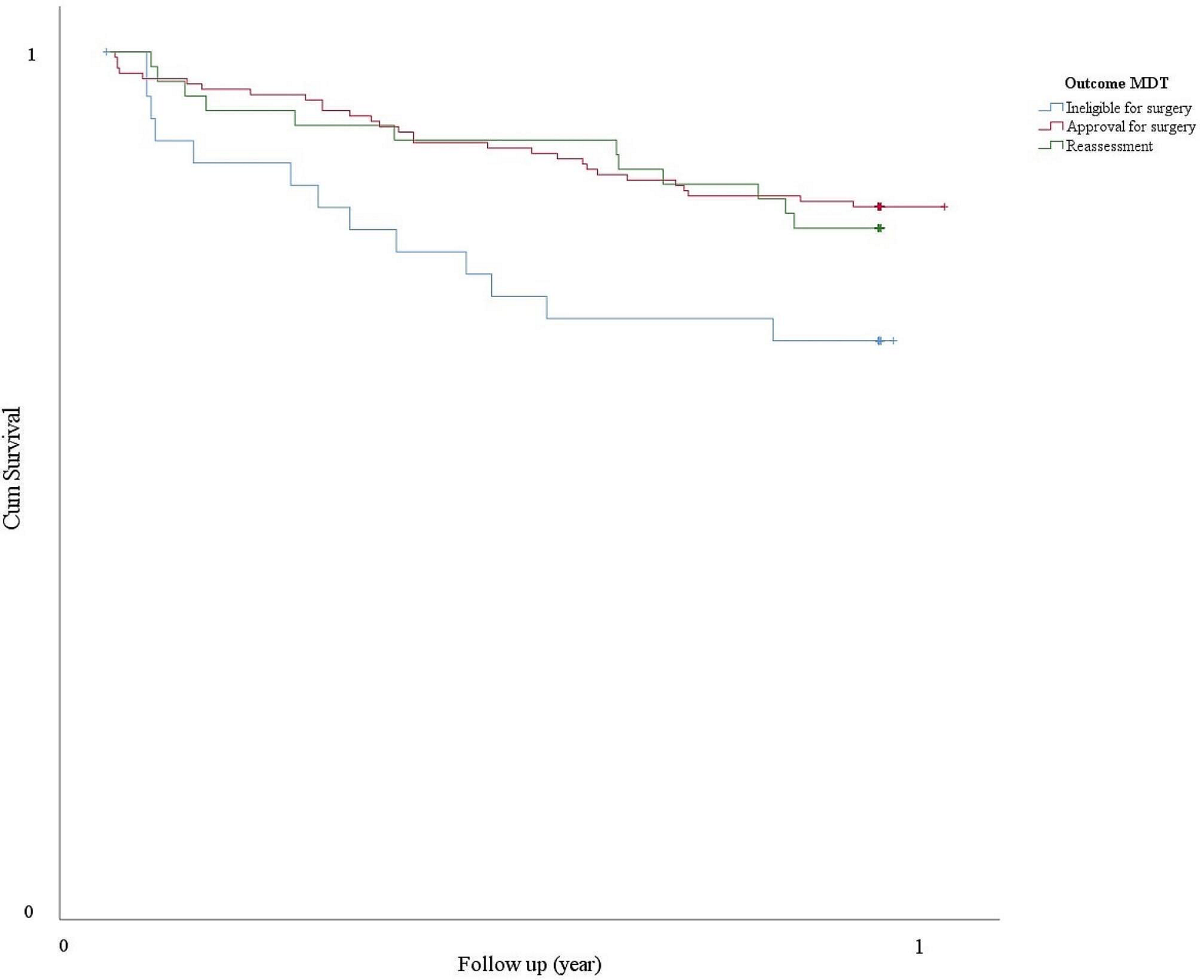
The Kaplan Meier curve of the one-year survival looking at the advice of the MDT assessment

Looking at the subdivision of the advice of the MDT meeting (approval for surgery, reassessment, or ineligibility for surgery), 31.8% (49/154), 32.1% (18/56), and 38.5% (15/39) patients, respectively, died during the one year follow-up period. The Kaplan-Meier curve is shown in Fig. 3.

## Discussion

We have described 249 patients discussed at a high-risk preoperative MDT assessment meeting in an academic and tertiary referral hospital in the Netherlands to illustrate the added value of MDT meetings for high-risk surgical patients. In total, 80.7% of patients were approved for surgery. The large percentage of approval, of which a substantial part is after reassessment, proves the willingness to operate on high-risk patients when, after thorough consideration, the risk-benefit ratio is considered acceptable.

Various instruments have been designed to support the preoperative assessment of patients, and prediction tools have been developed to improve the appraisal of the risk-benefit ratio for both patients and physicians [8, 19]. The NSQIP Surgical Risk Calculator developed by the ACS is a well-known example [8]. The ACS NSQIP surgical risk calculator is a validated tool used to counsel patients regarding their risks of postoperative complications of surgical treatment [20]. The use of risk assessment tools is twofold: first, they support decision-making because of their ability to estimate possible perioperative risks, which can then be discussed with patients. Second, they can alert physicians to a possible negative risk-benefit ratio in a particular patient [8].

When the population was stratified according to the ACS NSQIP surgical risk calculator for severe complications and death within 30 days after surgery, most of the population had an above-average risk of these complications, indicating the complexity and frailty of the patients included. Of the patients who underwent surgery in our cohort, 32.7% suffered from a major complication in the postoperative period, and 19% were deceased within the first year of follow-up.

The favorable clinical outcome of the patients approved by the MDT, compared to the ASC NSQIP surgical risk calculator, might be explained by the contribution of the MDT to the increased awareness of the vulnerability of these patients while at the same time providing the opportunity to reach multidisciplinary agreement on optimal preoperative, perioperative, and postoperative care. A major mechanism is a kind of Hawthorne effect [21] where the fact that a patient was selected for the MDT leads all clinical teams to spend more time and thought on the patient than other patients, such as a senior surgeon with a faster surgical time rather than a more unexperienced surgeon. However, innovations in the past few years in preoperative, perioperative, and postoperative care also need to be considered. The risk calculator was developed in 2013, and a retrospective cohort was selected from 2015 to 2020. Furthermore, the healthcare systems of the Netherlands and the United States of America are organized differently, so the use of risk calculators and MDT meetings may not be directly comparable.

A proportion of the patients who were reassessed went for additional consultation with, for instance, a cardiologist or pulmonologist, which could lead to either a different risk evaluation and/or different preoperative and perioperative management of these patients, possibly contributing to the reduction of complications. Furthermore, the contribution of a physiotherapist and/or dietician resulting in a multimodal prehabilitation program [22], might have also improved the outcome of these high-risk patients.

In our cohort study, four patients who underwent surgery while being considered ineligible for surgery died within the first year. This outcome suggests that the MDT can still select patients in which the disadvantages outweigh the potential benefit; however, because in three cases, the surgery was performed in an emergency setting, it is not possible to conclude this.

Another outcome that suggests the ability of the MDT to select the correct patients is the comparable one year mortality between patients who were approved for surgery (72.3%) and the patients in the group considered ineligible for surgery (65.9%). It is important to mention that the majority of patients had a non-oncological surgery. However, it is difficult to confirm this hypothesis because it is impossible to know what would have happened if the patient was not operated on or vice versa. Other explanations for the comparable one year mortality might be that some patients did not proceed with planned surgery but underwent less invasive treatment and that the indication for surgery was not vital in all patients and was therefore questioned.

These possible benefits cannot be demonstrated in the current observational study of a cohort of high-risk patients. However, the results of our cohort were comparable to those reported in the limited published literature. Vernooij et al. described in a retrospective cohort study that preoperative MDT meetings for noncardiac surgery led to a high rate of alterations to the initial surgical and anesthesia management plans, including a high percentage (43%) of decisions to convert the initially planned surgery to nonsurgical management [23].

Another Dutch retrospective cohort study concluded that implementation of a preoperative MDT meeting for frail patients with colorectal cancer improves the risk stratification and prehabilitation, resulting in comparable postoperative outcomes compared to non-frail patients [24]. Sroka et al. described the introduction of high-risk MDT meetings and reported a comparable one year mortality in the group of patients who underwent surgery after approval in the MDT of 18.7% [25].

A systematic review in 2008 showed that the overall incidence of in hospital adverse events was 9.2%, and almost half of these events were regarded as preventable. The authors concluded that funding and effort should be concentrated on interventions aimed at reducing these hospital adverse events [26]. Hospitals have been devoted to improving patient safety in the past few decades. An MDT meeting for high-risk patients is an excellent example of such an improvement and could be of increasing importance with the expected growing group of frail and multimorbid surgical patients combined with increasing surgical complexity. This cohort study shows the vulnerability and complexity of high-risk patients and shows that high-quality care can be administered with thorough planning and deliberation. Defining perioperative risk is complex and depends on the interaction between surgical, anesthetic, and patient-specific factors [27]. Furthermore, among physicians, the ability to recognize high-risk patients is highly variable [28]. Therefore, preoperative consultation by an anesthesiologist has been implemented in most national and international guidelines [29–32]. We believe that MDT meetings contribute to the personalized management of vulnerable patients and help to optimize their care. MDT meetings can act as a safety mechanism and provide the opportunity for questioning and aligning the practice of individuals. Especially in a tertiary hospital, where secondary opinions and complex patients are common, the opinions of different physicians can contribute to a personalized treatment plan. Sroka et al. also endorse the value of MDT meetings in their retrospective study on the development and implementation of an anesthesiologist-led multidisciplinary committee to evaluate high-risk surgical patients to improve surgical appropriateness [24]. In agreement with these investigators, we believe in the intrinsic value of MDT meetings in improving patient outcomes.

Our study has several limitations. The most significant limitation is the retrospective design, which is prone to selection bias and confounding factors. The admission for the MDT was based on the opinion of the pre-operative consulting anesthesiologist, several patients were selected for the MDT because of specific diseases with high-risk for anesthesia such as pulmonary hypertension or cardiac failure. On the other hand, many patients were selected based on a combination of factors and for them, there were no hard objective criteria. Also the choice of treatment and patient selection was based on local experience, this all may have resulted in selection bias. More extensive multicenter prospective studies are needed to quantify the impact of preoperative MDT discussions reliably and show the value of preoperative MDT meetings on patient outcomes. Furthermore, if the value of preoperative MDT meetings is proofed, there is a need for objective criteria which patient is selected for these meetings. The distinction between this study cohort and other studies was the heterogeneity of the cohort, which is comparable to the daily practice of every hospital. The frailty of the cohort was confirmed as 97.2% had an ASA classification of 3 or 4, and the mean CCI was above six. Furthermore, the calculated ESC/ESA risk score for the proposed procedure was intermediate or high in 88% of the patients. However, the heterogeneity of the cohort makes it difficult to draw conclusions about mortality. To fully answer the question of what value MDT meetings have for patients, an analysis of patients’ post-MDT quality of life and functional status is required. Implementing high-risk MDTs will also be easier when a cost-effective study shows a positive financial argument; therefore, this could be investigated in future studies. To prove the superiority of MDT meetings, a control group of patients comparable to the current cohort is needed. Unfortunately, at time of the design of this study we did not have a cohort available to compare with the study population. A randomized controlled trial might not be ethical; however, a prospective observational study might prove the added value of an MDT meeting for high-risk patients. We believe that MDT meetings contribute to personalized treatment and patient safety and should be endorsed. However, definite proof of the benefit of MDT meetings is still lacking.

## Conclusion

This cohort study shows the vulnerability and complexity of high-risk patients and highlights the opportunity of MDT meetings to optimize care for these patients by contributing to patient selection and providing the opportunity to reach multidisciplinary agreement on optimal preoperative, perioperative, and postoperative care.

### Electronic supplementary material

Below is the link to the electronic supplementary material.

**Supplementary Material 1:** Supplementary Tables A-D

## Data Availability

The datasets used and/or analysed during the current study are available from the corresponding author on reasonable request.

## Abbreviations

ACC/AHA: American College of Cardiology/American Heart Association
ACS: American College of Surgeons
ASA: American Society of Anesthesiology
CCI: Charlson Comorbidity Index
ESA: European Society of Anaesthesiology
ESC: European Society of Cardiology
ICU: Intensive Care Unit
IQR: Interquartile Range
MDT: Multidisciplinary Team
NSQIP: National Surgical Quality Improvement Program
WMO: Wet medisch-wetenschappelijk onderzoek met mensen
