# Supplementary material for: Does preoperative multidisciplinary team assessment of high-risk patients improve the safety and outcomes of patients undergoing surgery?
Source: BMC Anesthesiol. 2024 Jan 2;24:9. doi: 10.1186/s12871-023-02394-5 (PMC10759340; doi:10.1186/s12871-023-02394-5)
Supplement: Supplementary file 1 — Supplementary Material 1: Supplementary Tables A-D [file 12871_2023_2394_MOESM1_ESM.pdf]

Supplementary

Table A Overview grading according Clavien Dindo

Grade	Definition
Grade I	Any deviation from the normal postoperative course without the need for pharmacological treatment or surgical, endoscopic, and radiological interventions<br>Allowed therapeutic regimens are: drugs as antiemetics, antipyretics, analgetics, diuretics, electrolytes, and physiotherapy. This grade also includes wound infections opened at the bedside
Grade II	Requiring pharmacological treatment with drugs other than such allowed for grade I complications<br>Blood transfusions and total parenteral nutrition are also included
Grade III	Requiring surgical, endoscopic or radiological intervention
IIIa	Intervention not under general anesthesia
IIIb	Intervention under general anesthesia
Grade IV	Life-threatening complication (including CNS complications)* requiring IC/ICU management
IVa	Single organ dysfunction (including dialysis)
IVb	Multiorgan dysfunction
Grade V	Death of a patient

*Brain hemorrhage, ischemic stroke, subarachnoid bleeding, but excluding transient ischemic attacks.
CNS, central nervous system; IC, intermediate care; ICU, intensive care unit.

https://doi.org/10.1371/journal.pone.0222570.t001

Table B Preoperative consultation

	Positive advice MDT	Re-assessment	Negative advice MDT	p-value
**Preoperative consultation cardiologist**	82	42	18	*0.006*
**Preoperative consultation pulmonologist**	39	16	6	*0.315*
**Preoperative consultation geriatrician**	13	5	2	*0.763*

Table C. Reasoning first negative advice of the multidisciplinary team meeting

**Reasoning negative advice**	
Too many~~uch~~ morbidities or too frail	n = 20
Alternative surgery, less invasive	n = 7
In~~No~~ correct indication surgery	n = 5
~~Patient wish~~Patient refused surgery ~~not to operate~~	n = 3
Pre-operative analysis showed new co-morbidity	n = 2
Other treatment first	n = 1
Missing data	n = 1

Table D. Input ACS NSQIP surgical risk calculator

		**Positive advice MDT**	**Re-assessment**	**Negative advice MDT**	**p-value**
**Age group**	Under 65 year	51 (33.1%)	14 (25%)	6 (15.4%)	*0.024*
	65-74 year	53 (34.4%)	15 (26.8%)	14 (35.9%)	
	75-84 year	34 (22.1%)	20 (35.7%)	18 (46.2%)	
	85 year and older	16 (10.4%)	7 (12.5%)	1 (2.6%)	
**Sex**	Female	59 (38.3%)	25 (44.6%)	13 (33.3%)	*0.520*
	Male	95 (61.7%)	31 (55.4%)	26 (66.7%)	
**Emergency case**	Yes	0	0	0	
	No	154	56	39	
**Functional Status**	Independent	101 (65.6%)	26 (46.4%)	16 (41%)	*0.019*
	Partially dependent	41 (26.6%)	25 (44.6%)	18 (46.2%)	
	Total dependent	12 (7.8%)	5 (8.9%)	5 (12.8)	
**ASA**	Healthy patient	0	0	0	*0.001*
	Mild systemic disease	7	0	0	
	Severe systemic disease	93	31	11	
	Constant threat to life	54	25	28	
	Moribund	0	0	0	
	Yes	21 (13.6%)	9 (16.1%)	6 (15.4%)	*0.892*

**Steroid use for chronic condition**	No	133 (86.4%)	47 (83.9%)	33 (84.6%)	
**Ascites within 30 days prior to surgery**	Yes	1 (0.6%)	1 (1.8%)	2 (5.1%)	*0.138*
	No	153 (99.4%)	55 (98.2%)	37 (94.9%)	
**Systemic sepsis within 48 hours prior to surgery**	None	152 (98.7%)	54 (96.4%)	37 (94.9%)	*0.133*
	Sepsis	0	0	0	
	SIRS	1 (0.6%)	2 (3.6%)	2 (5.1%)	
	Septic shock	0	0	0	
**Ventilator dependent**	Yes	4 (2.6%)	2 (3.6%)	1 (2.6%)	*0.926*
	No	150 (97.4%)	54 (96.4%)	38 (97.4%)	
**Disseminated cancer**	Yes	16 (10.4%)	7 (12.5%)	4 (10.3%)	*0.911*
	No	136 (88.3)	49 (87.5%)	35 (89.7%)	
**Diabetes**	No	118 (76.6%)	42 (75%)	23 (59%)	*0.069*
	Yes	35 (22.7%)	14 (25%)	16 (41%)	
**Hypertension requiring medication**	Yes	117 (76.0%)	48 (85.7%)	30 (76.9%)	*0.339*
	No	36 (23.4%)	8 (14.3%)	9 (23.1%)	
**Congestive heart failure in 30 days prior to surgery**	Yes	60 (39%)	29 (51.8%)	19 (48.7%)	*0.193*
	No	94 (61%)	27 (48.2%)	20 (51.3%)	
**Dyspnea**	No	73 (47.4%)	16 (28.6%)	6 (15.4%)	*0.002*
	**With moderate exertion**	61 (39.6%)	30 (53.6%)	24 (61.5%)	
	**At rest**	20 (13%)	10 (17.9%)	9 (23.1%)	
	**Yes**	31 (20.1%)	9 (16.1%)	7 (17.9%)	*0.793*

Current smoker within 1 year	**No**	121 (78.6%)	46 (82.1)	32 (82.1%)	
	**Missing**	2	1		
History of Severe COPD	**Yes**	21 (13.6%)	10 (17.9)	5 (12.8%)	*0.715*
	**No**	132 (85.7%)	46 (82.1)	34 (87.2%)	
Dialysis	**Yes**	6 (3.9%)	2 (3.6%)	1 (2.6%)	*0.922*
	**No**	147 (95.5%)	54 (96.4%)	38 (97.4%)	
Acute Renal Failure	**Yes**	4 (2.6%)	2 (3.6%)	0	*0.291*
	**No**	149 (69.8%)	54 (96.4%)	39	
BMI		26.8 (SD 6.4)	26.2 (SD 6.6)	26.1 (SD 7.2)	*0.374*
	**High**	169.8 (SD 15.4)	171.9 (SD 10.9)	171 (SD 8.6)	*0.008*
	**Weight**	80 (SD 22.1)	78.8 (SD 22.1)	77 (SD 25.2)	*0.133*
